# Intracoronary versus Intravenous Administration of Abciximab in Patients with Acute Coronary Syndrome: A Meta-Analysis

**DOI:** 10.1371/journal.pone.0058077

**Published:** 2013-02-28

**Authors:** Jie-Ning Wang, Shu Diao, Yuan-Jun Tang, An-Ji Hou, Hai-Bo Yuan, Yan Zheng, Yu-Hao Zhou

**Affiliations:** 1 Director of the Hospital, Shanghai Seventh People’s Hospital, Shanghai, China; 2 Vice Director of the Hospital, Shanghai Seventh People’s Hospital, Shanghai, China; 3 Department of Pharmaceutical Analysis, School of Pharmacy, Second Military Medical University, Shanghai, China; 4 Department of Oncology, Shanghai Seventh People’s Hospital, Shanghai, China; 5 Department of Science, Shanghai Seventh People’s Hospital, Shanghai, China; 6 Department of Rehabilitation Institute, Shanghai Seventh People’s Hospital, Shanghai, China; Cliniche Humanitas Gavazzeni, Italy

## Abstract

**Background:**

Abciximab is a widely used adjunctive therapy for acute coronary syndrome (ACS). However, the effect of intracoronary (IC) administration of abciximab on cardiovascular events remains unclear when compared with intravenous (IV) therapy.

**Methodology and Principal Findings:**

We systematically searched the Medline, Embase, and Cochrane Central Register of Controlled Trials databases and reference lists of articles and proceedings of major meetings for obtaining relevant literature. All eligible trials included ACS patients who received either IC administration of abciximab or IV therapy. The primary outcome was major cardiovascular events, and secondary outcomes included total mortality, reinfarction, and any possible adverse events. Of 660 identified studies, we included 9 trials reporting data on 3916 ACS patients. Overall, IC administration of abciximab resulted in 45% reduction in relative risk for major cardiovascular events (RR; 95% confidence interval [CI], 24−60%), 41% reduction in RR for reinfarction (95% CI, 7−63%), and 44% reduction in RR for congestive heart failure relative to IV therapy (95% CI, 8−66%); however, compared to IV therapy, IC administration of abciximab had no effect on total mortality (RR, 0.69; 95% CI, 0.45−1.07). No other significant differences were identified between the effect of IC abciximab administration and IV therapy.

**Conclusions/Significance:**

IC administration of abciximab can reduce the risk of major cardiovascular events, reinfarction, and congestive heart failure when compared with IV therapy.

## Introduction

Previous trials have indicated that the management of acute coronary syndrome (ACS) is undergoing major changes [1]. Percutaneous coronary intervention (PCI) for recanalization of related infarcted arteries is considered the most effective therapy for ACS [2], [3]. Nearly all trials reported about the efficacy and safety of intravenous (IV) administration of abciximab. Recently, several studies suggested another effective therapy, which demonstrated that intracoronary (IC) administration of abciximab with high local concentrations of the antibody might be favorable for the dissolution of thrombi and microemboli, which is associated with faster recovery of the myocardial microcirculation and better prognosis [4]–[5].

Several randomized controlled trials argued that rapidly active abciximab should be used in the ambulance, which contributed to faster recovery and increased efficacy in ACS patients [6]–[9]. However, few studies have provided evidence about the differences in efficacy and safety between IC and IV administration of abciximab for clinical practice, which makes interpretation of the results difficult for clinicians. A previous meta-analysis [10] compared IC and IV administration of glycoprotein IIb/IIIa inhibitors in patients with ACS. This study included both abciximab and several other drugs, including tirofiban and eptifibatide, which restricted us to evaluate the efficacy and safety of abciximab.

Recently, several large-scale randomized controlled trials have investigated IC abciximab administration [11], [12]. Data from these recent trials needed to be re-evaluated and combined with the data from previous literature on IC abciximab administration. Therefore, we conducted a systematic review and meta-analysis of pooled data from randomized controlled trials to evaluate the possible effect of IC administration of abciximab compared with IV therapy, on cardiovascular outcomes in ACS patients.

## Methods

### Data Sources, Search Strategy, and Selection Criteria

We systematically searched the English literature to identify all relevant randomized, controlled trials regardless of publication status (published, unpublished, in press, or in progress). Relevant trials were identified using the following procedure:

1. Electronic searches: We searched the Medline, Embase, and Cochrane Central Register of Controlled Trials electronic databases for articles published through May 10, 2012, using “intracoronary” OR “intravenous” AND “abciximab” AND “randomized controlled trials” OR “clinical trials” as the search terms.
2. Other sources: We searched ongoing randomized controlled trials in the metaRegister of Controlled Trials, which lists trials that are registered as completed but not yet published. Furthermore, we reviewed bibliographies of publications for potentially relevant articles. Medical subject headings, methods, patient population, interventions, and outcome variables of these studies were used to identify relevant trials. This review was conducted and reported according to the Preferred Reporting Items for Systematic Reviews and Meta-Analysis (PRISMA) Statement issued in 2009 (Table S1) [13].

The literature search was undertaken independently by 2 authors (Hai-Bo Yuan and Yan Zheng) with a standardized approach. Any inconsistencies between these 2 authors were settled by the primary author (Yu-Hao Zhou) until a consensus was reached. We restricted our research to randomized controlled trials, which were less likely to be subject to confounds and bias than observational studies. Studies were identified for inclusion if: (1) the study was a randomized controlled trial; (2) the trial assessed the effects of IC administration of abciximab compared with IV therapy; (3) the duration of follow-up was at least 30 days; and (4) the trial reported at least 1 outcome of major cardiovascular events, total mortality, reinfarction, or 3 of the aforementioned events.

### Data Collection and Quality Assessment

Two investigators (Yuan-Jun Tang and Yan Zheng) independently extracted and collected data using a standardized data extraction protocol. Any discrepancy was settled by group discussion, and then, the primary author (Yu-Hao Zhou) made the final decision. The data collected included baseline patient characteristics (first author or study group’s name, publication years, number of patients, mean age, percentage of males, patient diseases, interventions, and the duration of follow-up. The outcomes investigated included major cardiovascular events, total mortality, reinfarction, target vessel revascularization (TVR), cardiac death, congestive heart failure, major bleeding, and stroke. Study quality was assessed using the Jadad scores [14] (Shu Diao and Jie-Ning Wang), which are based on the 5 following subscales: randomization (1 or 0), concealment of the treatment allocation (1 or 0), blinding (1 or 0), completeness of follow-up (1 or 0), and the use of intention-to-treat analysis (1 or 0). A “score system” (ranging from 0 to 5) has been developed for assessment. In our study, we considered a study awarded a score of 4 or above to be a high-quality study.

### Statistical Analysis

We allocated the results of each randomized controlled trial as dichotomous frequency data. Individual study relative risks (RRs) and 95% confidence intervals (CIs) were calculated from event numbers extracted from each trial before data pooling. The overall RR and 95% CI of serious vascular events and any possible adverse events were also calculated. Heterogeneity of the treatment effects between studies was investigated visually by scatter plot analysis and statistically using the heterogeneity I^2^ statistic [15]. To explore potential heterogeneity in estimates of treatment effect, we performed a sensitivity analysis and subgroup analysis to eliminate the intrinsic differences among included trials. Although the fixed-effects and random-effects models yielded similar conclusions, we chose to use the random-effects model, which assumed that the true underlying effect varied among included trials. Moreover, many investigators consider the random-effects model to be a more natural choice than the fixed-effects model in medical decision-making contexts [16], [17]. Egger’s test [18] was used to check for potential publication bias. All reported P values are 2-sided, and values with P<0.05 were regarded as statistically significant for all included studies. Statistical analyses were performed using STATA (version 10.0).

## Results

**Figure 1:**
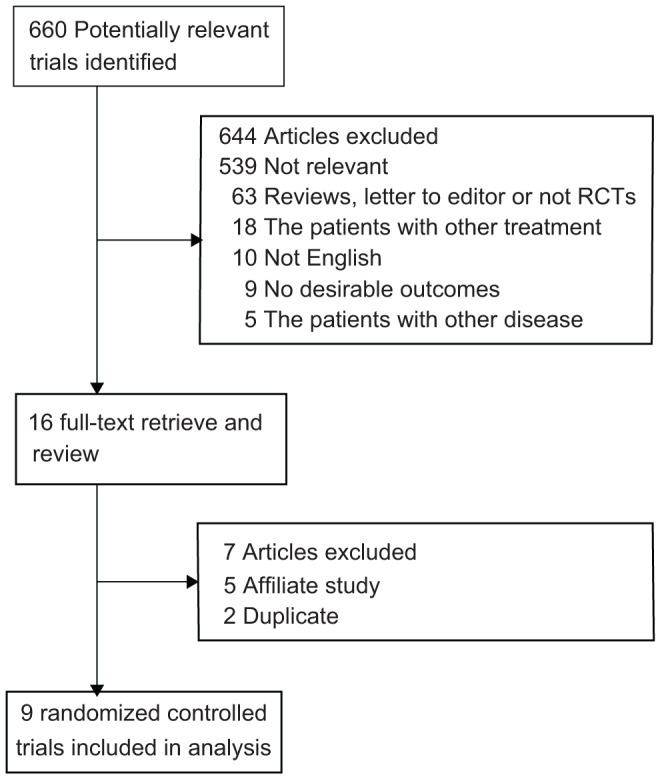
Flow diagram of the literature search and trials selection process.

We identified 660 articles from our initial electronic search, of which 644 were excluded during an initial review (title and abstract). We retrieved the full text for the remaining 16 articles, and 9 randomized controlled trials met the inclusion criteria (Figure 1 and Figure S1), which consisted of data from 3916 ACS patients. Table 1 summarizes the characteristics of these trials and the important baseline information of the included patients. The trials included in this study compared IC administration of abciximab with IV therapy. Five of these studies [11], [12], [19]–[21] compared IC to IV therapy in patients with ST-elevation myocardial infarction (STEMI), and the other 4 trials [5], [22]–[24] evaluated individuals with ACS. The mean age of the patients ranged from 57 to 68, the patient follow-up duration ranged from 1 to 12 months, and the number of patients included in each study ranged from 45 to 2065. The outcomes were major cardiovascular events available in 6 trials [5], [12], [19], [20], [22], [23], total mortality in 9 trials [5], [11], [12], [19]–[24], reinfarction in 7 trials [5], [11], [12], [19]–[22], TVR in 5 trials [5], [12], [19], [20], [22], cardiac death in 3 trials [11], [12], [23], congestive heart failure in 2 trials [11], [20], major bleeding in 4 trials [11], [19], [21], [22], and stroke in 2 trials [11], [23]. Although the included trials scarcely reported on the key indicators of trial quality, the quality of the trials was also assessed according to the pre-defined criteria using Jadad scores [14]. Overall, 3 trials [5], [11], [12] scored 4, 3 trials [20], [21], [23] scored 3, and the remaining 3 trials [19], [22], [24] scored 2.

**Table 1:** Design and baseline characteristic of trials included in the systematic review and meta-analysis.

Source	No. of patients	Mean age, y	Sex (male)	Disease	Intervention	follow-up (months)	Reporting Outcomes	Jaded score
H Thiele 2012 [11]	2065	63	75%	STEMI	IC abciximab (0.25 mg/kg); IV abciximab(0.25 mg/kg)	3	mortality, reinfarction	4
AZ Iversen 2011 [19]	355	62	81%	STEMI	IC abciximab (0.25 mg/kg); IV abciximab(0.25 mg/kg)	12	mortality, reinfarction	2
YL Gu 2010 [12]	534	64	74%	STEMI	IC abciximab (0.25 mg/kg); IV abciximab(0.25 mg/kg)	1	mortality, reinfarction	4
H Thiele 2008 [20]	154	65	80%	STEMI	IC abciximab (0.25 mg/kg); IV abciximab(0.25 mg/kg)	1	mortality	3
J Wöhrle 2003 [5]	403	60	79%	ACS	IC abciximab (20 mg); IV abciximab(20 mg)	1	mortality, reinfarction	4
Alberto DR 2009 [21]	50	68	76%	STEMI	IC abciximab (0.25 mg/kg); IV abciximab(0.25 mg/kg)	1	mortality, reinfarction	3
AK Kakkar 2004 [22]	173	57	75%	ACS	IC abciximab (0.25 mg/kg); IV abciximab(0.25 mg/kg)	6	mortality, reinfarction	2
JG Galache Osuna 2006 [23]	137	62	82%	ACS	IC abciximab (0.25 mg/kg); IV abciximab(0.25 mg/kg)	6	mortality	3
F Bellandi 2004 [24]	45	62	78%	AMI	IC abciximab (0.25 mg/kg); IV abciximab(0.25 mg/kg)	1	mortality	2

**Figure 2:**
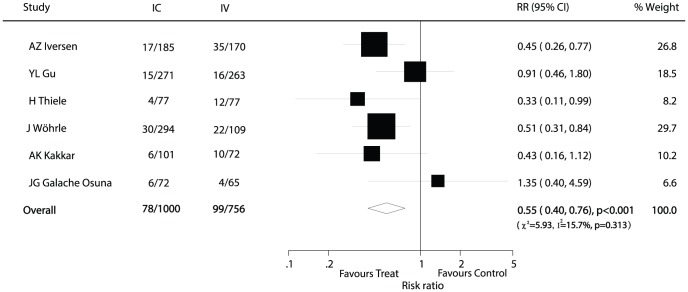
Effects of IC administration of abciximab on risk of major cardiovascular events as compared to IV therapy in patients with ACS.

Data for the effect of IC abciximab administration on major cardiovascular events were available from 6 trials [5], [12], [19], [20], [22], [23], which included 1756 patients and reported 177 serious vascular events (Figure 2). Overall, the pooled RR value indicated that IC abciximab therapy was associated with a clinically and statistically significant reduced risk of major cardiovascular events when compared with IV therapy (RR, 0.55; 95% CI, 0.40−0.76, with unimportant heterogeneity).

**Figure 3:**
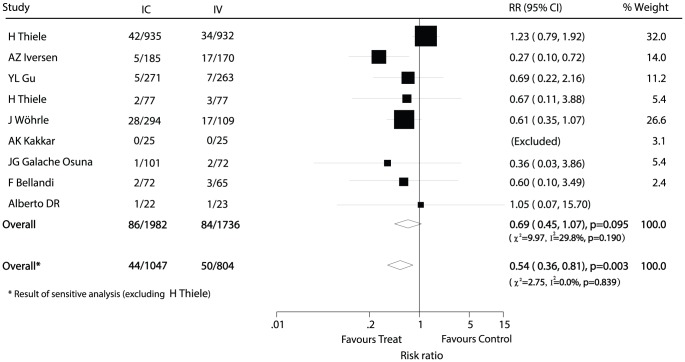
Effects of IC administration of abciximab on risk of total death as compared to IV therapy in patients with ACS.

Data for the effect of IC administration of abciximab on total mortality were available from 9 trials [5], [11], [12], [19]–[24], including 3718 patients and 170 events of total mortality. Overall, IC abciximab administration reduced the risk of mortality by 31% but was not associated with a statistically significant reduction in the risk of mortality events (RR, 0.69; 95% CI, 0.45−1.07; Figure 3). We noted that there was some evidence of heterogeneity across the included studies; therefore, sensitivity analyses were performed to explore any possible intrinsic reason for this finding. We then excluded the trial of Thiele et al [11], which specifically included a large number of patients that may have contributed a large weight to the pooled conclusion. After this exclusion, we concluded that compared to IV therapy, IC administration of abciximab was associated with a reduction in the total mortality risk, which decreased by 46% (RR, 0.54; 95% CI, 0.36−0.81; Figure 3).

**Figure 4:**
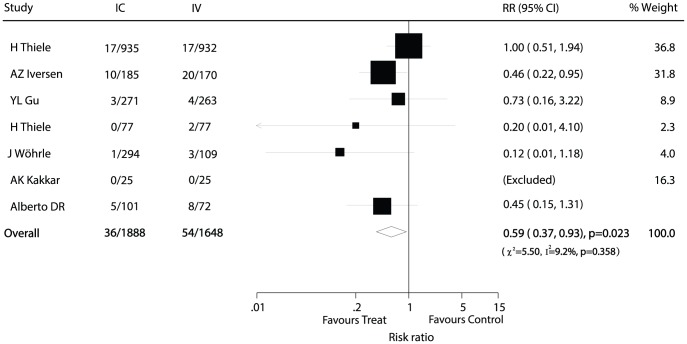
Effects of IC administration of abciximab on risk of reinfarction as compared to IV therapy in patients with ACS.

Data for the effect of IC abciximab administration on reinfarction were available from 7 trials [5], [11], [12], [19]–[22], which included 3536 patients and reported 90 reinfarction events. Overall, we noted that IC administration of abciximab resulted in a 41% reduction in the risk of reinfarction when compared with IV therapy (RR, 0.59; 95% CI: 0.37−0.93, with unimportant heterogeneity; Figure 4).

Five of the trials [5], [12], [19], [20], [22] included 1619 patients with 96 TVR events. There was no evidence to show that IC abciximab administration protected against TVR risk (RR, 0.64; 95% CI, 0.32−1.29; Table 2), although heterogeneity was observed in the magnitude of the effect across the included trials. However, after sequential exclusion of each trial from all pooled analyses, the results were not affected by exclusion of any specific trial.

**Table 2:** Summary of the relative rates with its 95%CI of all outcomes assessed.

Outcomes	IC group	IV group	RR and 95%CI	P value	heterogeneity	P value for heterogeneity
Major cardiovascular event	78/1000	99/756	0.55 [0.40, 0.76]	<0.001	16%	0.31
Total mortality	86/1982	84/1736	0.69 [0.45, 1.07]	0.10	30%	0.19
reinfarction	36/1888	54/1648	0.59 [0.37, 0.93]	0.02	9%	0.36
TVR	44/928	52/691	0.64 [0.32, 1.29]	0.21	56%	0.06
Cardiac death	46/1278	41/1260	1.12 [0.74, 1.69]	0.60	0%	0.65
Congestive heart failure	24/1012	43/1012	0.56 [0.34, 0.92]	0.02	0%	0.67
Major bleeding	35/1296	31/1266	1.00 [0.57, 1.74]	1.00	11%	0.34
stroke	6/1057	9/1064	0.66 [0.24, 1.86]	0.43	0%	0.73

The effect of IC administration of abciximab on the risk of cardiac death was reported in 3 trials [11], [12], [23], which included 2538 patients and recorded 87 cardiac death events. Overall, there was no effect of IC abciximab administration on the risk of cardiac death compared with IV therapy (RR, 1.12; 95% CI, 0.74−1.69, with unimportant heterogeneity; Table 2).

The risk of congestive heart failure was reported in 2 trials [11], [20], including 2024 individuals and 67 events of congestive heart failure. Overall, IC administration of abciximab reduced the risk of congestive heart failure by 44% when compared with IV therapy (RR, 0.56; 95% CI, 0.34−0.92, with unimportant heterogeneity; Table 2).

Four trials [11], [19], [21], [22] reported the effect of IC administration of abciximab on major bleeding, which included 2562 patients and recorded 66 major bleeding events. No effect of IC abciximab administration on the risk of major bleeding was observed (RR, 1.00; 95% CI, 0.57−1.74, with unimportant heterogeneity; Table 2).

Of the 9 trials included in our meta-analysis, only 2 provided data about stroke events [11], [23], which included 2121 patients and reported 15 stroke events. Overall, the pooled analysis showed no significant differences between IC abciximab administration and IV therapy for stroke (RR, 0.66; 95% CI, 0.24−1.86, with unimportant heterogeneity; Table 2).

Subgroup analyses were conducted for major cardiovascular events and total mortality. We noted that IC administration of abciximab was associated with a reduction in the risk of major cardiovascular events when the number of patients was more than 200, the mean age of patients was below 63, the proportion of men was less than 80%, or the Jadad score was less than 4. Similarly, when compared with IV therapy, IC administration of abciximab showed a clear effect on total mortality when the mean age of patients was below 63, the proportion of men was greater than 80%, or the Jadad score was less than 4. No other significant differences were identified between the effect of IC abciximab administration and IV therapy, when based on additional subset factors (Table 3).

**Table 3:** Subgroup analysis for the effect of IC versus IV therapy on major cardiovascular events, and mortality.

Outcomes	Group	event/total patients		Relative risk (RR)	P value	heterogeneity	P value for heterogeneity
		IC group	IV group				
**Major cardiovascular event**	**Published years**						
	After 2008	36/533	63/510	0.54 [0.31, 0.95]	0.03	42%	0.18
	Before 2008	42/467	36/246	0.57 [0.34, 0.95]	0.03	19%	0.29
	**Number of** **patients**						
	>200	62/750	73/542	0.56 [0.38, 0.82]	0.003	28%	0.25
	<200	16/250	26/214	0.55 [0.25, 1.20]	0.13	37%	0.21
	**Mean age**						
	63 or more	19/348	28/340	0.60 [0.23, 1.59]	0.31	58%	0.12
	Less than 63	59/652	71/416	0.51 [0.37, 0.71]	<0.001	0%	0.42
	**Gender (sex)**						
	80% or more	27/334	51/312	0.53 [0.27, 1.02]	0.06	39%	0.20
	Less than 80%	51/666	48/444	0.59 [0.39, 0.90]	0.01	14%	0.31
	**Diseases**						
	STEMI	36/533	63/510	0.54 [0.31, 0.95]	0.03	42%	0.18
	ACS	42/467	36/246	0.57 [0.34, 0.95]	0.03	19%	0.29
	**Follow-up**						
	30 days	49/642	50/449	0.58 [0.35, 0.94]	0.03	32%	0.23
	More than 30 days	29/358	49/307	0.54 [0.30, 0.97]	0.04	29%	0.24
	**Jadad score**						
	4 or 5	45/565	38/372	0.65 [0.37, 1.15]	0.14	46%	0.17
	<4	33/435	61/384	0.49 [0.31, 0.76]	0.002	10%	0.34
	**Overall**	78/1000	99/756	0.55 [0.40, 0.76]	<0.001	16%	0.31
**Mortality**	**Published years**						
	After 2008	54/1493	61/1467	0.67 [0.30, 1.49]	0.32	63%	0.04
	Before 2008	32/489	23/269	0.61 [0.36, 1.01]	0.06	0%	0.95
	**Number of** **patients**						
	>200	80/1685	75/1474	0.67 [0.36, 1.25]	0.20	68%	0.02
	<200	6/297	9/262	0.61 [0.22, 1.70]	0.35	0%	0.95
	**Mean age**						
	63 or more	49/1308	44/1297	1.11 [0.74, 1.66]	0.61	0%	0.55
	Less than 63	37/674	40/439	0.51 [0.32, 0.80]	0.004	0%	0.65
	**Gender (sex)**						
	80% or more	9/334	23/312	0.37 [0.17, 0.81]	0.01	0%	0.57
	Less than 80%	77/1648	61/1424	0.87 [0.59, 1.28]	0.49	13%	0.33
	**Diseases**						
	STEMI	54/1493	61/1467	0.67 [0.30, 1.49]	0.32	63%	0.04
	ACS	32/489	23/269	0.61 [0.36, 1.01]	0.06	0%	0.95
	**Follow-up**						
	30 days	36/689	28/497	0.64 [0.40, 1.03]	0.07	0%	0.98
	More than 30 days	50/1293	56/1239	0.59 [0.22, 1.56]	0.29	65%	0.03
	**Jadad score**						
	4 or 5	75/1500	58/1304	0.86 [0.51, 1.45]	0.57	50%	0.14
	<4	11/482	26/432	0.40 [0.20, 0.81]	0.01	0%	0.80
	**Overall**	86/1982	84/1736	0.69 [0.45, 1.07]	0.10	30%	0.19

We used Egger’s test [18] to check for potential publication bias, which showed no evidence of publication bias for the outcomes of major cardiovascular events (P = 0.681), total mortality (P = 0.258), and reinfarction (P = 0.164).

## Discussion

This comprehensive, quantitative review included 3916 patients from 9 trials with a broad range of baseline characteristics. Although the studies included clinically diverse populations (with STEMI or ACS), there was little heterogeneity among results of different trials, and the results were stable when subjected to various sensitivity analyses.

Our study indicated that IC administration of abciximab produced a 45% reduction in major cardiovascular events compared with IV therapy. In addition, IC abciximab administration played an important role in the incidence of reinfarction. However, IC administration of abciximab was not associated with a statistically significant decrease in the risk of total mortality. Finally, we noted that major bleeding events were less markedly reduced in patients who underwent IC administration of abciximab than those given IV therapy during the active study periods.

Our main findings are in contrast with the findings of previous research [25], and our findings also support the conclusion that IC administration of abciximab has no significant effect on the risk of total mortality and major bleeding. However, we concluded that IC administration of abciximab had a clear effect on the risk of major cardiovascular events and reinfarction. Furthermore, sensitivity analysis indicated that IC abciximab administration might play an important role in total death when compared with IV therapy. Finally, we also performed subgroup analyses based on several important factors.

The subgroup analyses indicated that IC administration of abciximab had no effect on major cardiovascular events when the number of patients was less than 200, the mean age of patients was above 63, the proportion of men was greater than 80%, or the study was high-quality (Jadad score 4 or 5) as compared with IV therapy. The reasons for this lack of difference are as follows: (1) a small sample size always contributed to broad confidence intervals; (2) older patients always had a poor prognosis; and (3) several other factors might play an important role in ACS pathogenesis and affect the efficacy of treatment, including smoking, alcohol abuse, and others, which occurred more frequently in male patients. Similarly, the results of the subgroup analyses were also supported when the mean age of the patients was below 63, and the proportion of men was greater than 80%, and low-quality (Jadad score less than 4) trials indicated that the risk of total mortality was significantly reduced by IC administration of abciximab when compared with IV therapy. Several of the reasons for these findings are the same as those mentioned above. In addition, in the patient subset with a proportion of men that was greater than 80%, the high incidence of reinfarction and low mortality rate might play important roles in the total mortality risk.

There were significant differences between IC administration of abciximab and IV therapy for major cardiovascular events and reinfarction; moreover, sensitivity analysis indicated that IC administration of abciximab produced a protective effect on total mortality. The reason for this could be that local doses of IC-administered abciximab might facilitate the diffusion of the antibody to platelets inside flow-limiting thrombi, resulting in improved dissolution of thrombi and microemboli at the culprit lesion and in the distal microcirculation [4], [23], [26], [27]. Therefore, IC abciximab administration had direct beneficial effects on major cardiovascular events in ACS patients.

IC abciximab administration played an important role in reducing the risk of congestive heart failure, which may be due to the pathophysiological rationale of improved perfusion and the reduction in infarct size by IC administration of abciximab. Furthermore, because information regarding the effects on infarct size, ST-segment resolution, or left ventricular ejection fraction were not available, this difference in congestive heart failure might be attributable to chance [11], [28].

No other significant differences were detected between IC administration of abciximab and IV therapy on the risk of TVR, cardiac death, major bleeding, and stroke. The reason for these absences of differences could be that these data provided by relatively few trials led us to be unable to obtain a reliable conclusion.

Previous trials indicated that the effect of IC abciximab administration on cardiovascular outcomes was better than that of IV therapy, whereas other outcomes provided a negative conclusion [11]. Our meta-analysis also supported the conclusion that IC administration of abciximab had a clear effect on major cardiovascular events; in addition, we concluded that IC abciximab administration played an important role in reinfarction, and might have an effect on total mortality. This study is promising due to the comprehensiveness of the available data and the broad range of clinically important features of ACS patients.

The limitations of our study are as follows: (1). Relatively few trials reported the results of IC administration of abciximab in patients with ACS. Although subgroup analyses suggested that IC abciximab administration was associated with statistically significant reductions in the risk of major cardiovascular events and total mortality in several subsets, these conclusions were variable because the number of trials included was few that restricted us from exploring the intrinsic effect. (2). Different PCI techniques might provide a biased view of the study question. (3). Inherent assumptions are made for any meta-analysis, the analysis used pooled data either published or provided by individual study authors, and individual patient data or original data were unavailable, which restricted us from performing a more detailed relevant analysis and obtaining more comprehensive results.

In conclusion, the findings of our study indicated that IC administration of abciximab produced a significant reduction in the risk of major cardiovascular events and reinfarction. Furthermore, it this treatment might also play an important role in the risk of total mortality in ACS patients. In future research, it will be important to focus on the patients’ baseline characteristics to provide patients the most suitable treatments. We suggest that the ongoing trials should be improved as follows: (1) the adverse effect events of trials should be recorded and reported normatively, particularly for serious adverse events, and the side-effects of treatment should be evaluated in any future trial; and (2) more attention should be paid to the role of treatment duration and dosage and to exploring the optimal dose and the duration of treatment.

## Supporting Information

Figure S1 **PRISMA Flowchart.**
(DOC)

Table S1 **PRISMA Checklist.**
(DOC)
