# Supplementary material for: Intracoronary versus Intravenous Administration of Abciximab in Patients with Acute Coronary Syndrome: A Meta-Analysis
Source: PLoS One. 2013 Feb 28;8(2):e58077. doi: 10.1371/journal.pone.0058077 (PMC3585408; doi:10.1371/journal.pone.0058077)
Supplement: Figure S1 — PRISMA Flowchart. (DOC) [file pone.0058077.s001.doc]

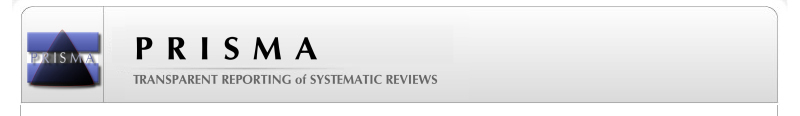
**PRISMA 2009 Flow Diagram**

**Screening**

**Included**

**Eligibility**

**Identification**

660 Potentially relevant articles identified in database searches

644 Article excluded

539 Not relevant

63 Reviews, letter to editor or not RCTs

18 The patients with other treatment

10 Not English

9 No desirable outcomes

5 The patients with other disease

16 full-text retrieve and review

7 Articles excluded

5 Affiliated trials

2 Duplicate studies

9 Articles included in meta-analysis
